# Indigenous Peoples’ rights in national climate governance: An analysis of Nationally Determined Contributions (NDCs)

**DOI:** 10.1007/s13280-023-01922-4

**Published:** 2023-10-11

**Authors:** Rosario Carmona, Graeme Reed, James Ford, Stefan Thorsell, Rocío Yon, Francisca Carril, Kerrie Pickering

**Affiliations:** 1 Department for the Anthropology of the Americas, University of Bonn, Bonn, Germany; 2 Center for Integrated Disaster Risk Management (CIGIDEN), Santiago, Chile; 3 Centre for Indigenous Knowledges, York University, Toronto, Canada; 4 Priestley International Centre for Climate, University of Leeds, Leeds, UK; 5 International Work Group for Indigenous Affairs (IWGIA), Copenhagen, Denmark; 6 Free University of Berlin, Berlin, Germany; 7 Center for Intercultural and Indigenous Research (CIIR), Santiago, Chile; 8 Environmental Sustainability Research Centre, Brock University, St. Catharines, Canada

**Keywords:** Climate governance, Climate policy, Indigenous Peoples, Indigenous Peoples’ rights, Nationally Determined Contributions (NDCs)

## Abstract

Although the recognition of Indigenous Peoples’ contributions to climate governance by the international community has gradually increased, a rights-based approach in national climate action is still largely absent. This article analyses the recognition of Indigenous Peoples’ rights in Nationally Determined Contributions (NDCs) under the Paris Agreement. We conducted a content analysis of all NDCs submitted between 2016 and May 2022. Through a five-pronged framework of sustainable self-determination, we assessed how the NDCs recognise: i. Indigenous Peoples as rights-holders; ii. Indigenous jurisdiction over land; iii. Indigenous knowledge systems; iv. Indigenous Peoples’ right to full and effective participation in climate governance; and v. the legacy of colonialism. NDCs with references related to Indigenous Peoples are increasing. However, questions remain regarding their sincerity and commitment to implementation. States must therefore make more significant efforts to ensure that the NDCs take a rights-based approach and contribute to strengthening Indigenous Peoples’ role and say in climate governance.

**Supplementary Information:**

The online version contains supplementary material available at 10.1007/s13280-023-01922-4.

## Introduction

Indigenous Peoples have been urgently warning society of disastrous climate change impacts (Whyte 2020) whilst also calling for deeper reflection on the underlying causes of climate change, which, beyond being attributed to the burning of fossil fuels, is the product of a mindset that justified colonialism (Cameron et al. 2021; Redvers et al. 2022) and the institutions and legal structures that have perpetuated it (Quijano 2000). The legacy of colonialism has not only increased Indigenous Peoples’ vulnerability to climate change, but also subjected them to climate policies that violate their individual and collective rights, affirmed in the UN Declaration on the Rights of Indigenous Peoples (UNDRIP) (Whyte 2017; Indigenous Climate Action 2021; Reed et al. 2021). To tackle this inequity, Indigenous Peoples have integrated climate change into their historical advocacy for self-determination. Aiming to achieve decision-making power in the climate negotiations under the UN Framework Convention on Climate Change (UNFCCC), they have demanded the respect of their rights, including their right to participation (Comberti et al. 2019; Sherpa 2019; Rashidi and Lyons 2021). Indigenous Peoples have also pushed for their legal and knowledge systems to be considered when designing and implementing climate action, so that measures affecting their territories align with their values, customary institutions, aspirations and needs.

Indigenous Peoples saw the result of this advocacy in 2015, securing the inclusion of a rights-based language in the preamble to the Paris Agreement and five other references to Indigenous Peoples, including the recognition of their knowledge (article 7 par. 5). The UNFCCC has amplified their demands (UNFCCC 2016), and following COPs have encouraged the participation of Indigenous Peoples and the consideration of their rights and knowledge systems in national climate governance (IIPFCC and CIEL 2021). Recognition in national and international climate policy agreements is the first important step, and in many countries, a vital prerequisite for national Indigenous Peoples’ movements to be able to hold states accountable and demand compliance with the rights of Indigenous Peoples in implementation.

Indigenous Peoples, however, continue to face multiple barriers to their effective engagement (Belfer et al. 2019; Shawoo and Thornton 2019), and their impact on international climate negotiations remains restricted (Tormos-Aponte 2021). The inclusion of Indigenous Peoples continues to depend on the will of State actors—upholding the ‘party-driven’ process—who do not recognise their right to self-determination (Gustafsson and Schilling-Vacaflor 2022; Shea and Thornton 2019). At the national level, safeguards protecting Indigenous Peoples’ rights tend to be approached by states as bureaucratic requirements to access climate funding; Indigenous Peoples continue to receive top-down proposals that restrict their meaningful engagement (Carmona 2023). Furthermore, states mainly include Indigenous knowledge aligning with institutional objectives, generating multiple tensions around problem definition and stakeholders legitimation (Petzold et al. 2020). These challenges can mainly be attributed to inadequate consideration of Indigenous Peoples as sovereign nations (Reo et al. 2017).

Despite the above, there is ample evidence that collaboration with Indigenous Peoples improves climate policy outcomes (IPCC 2022a, b). With the Paris Agreement promoting a more polycentric climate governance (Beck et al. 2022), this collaboration presents an opportunity for states, which cannot address climate change in isolation (Sayer et al. 2013). Indigenous Peoples have expressed their willingness to collaborate and have demanded that states include them in national climate governance and, specifically, in the definition and implementation of Nationally Determined Contributions (NDCs) (Facilitative Working Group 2021b; IIPFCC 2022). NDCs are the mechanism the Paris Agreement requires (art. 4) to develop medium-term, country-driven action plans grounded within bounded self-differentiation (Pauw and Klein 2020). Despite criticism of their voluntary nature (Geden 2016), NDCs are currently considered a ‘keystone of the international climate policy process’ (Pauw and Klein 2020, p. 405). Parties to the UNFCCC are developing guidelines and mechanisms to make the process more accountable, amongst them a 'transparency framework' that aims to standardise the metrics, priorities, and communication of NDCs (Kuyper et al. 2018). This process is complemented by a Global Stocktake every 5 years, the first of which is set to be completed at the end of 2023.

The NDCs, therefore, represent a standardised mechanism for identifying country priorities from a global perspective (Shea and Thornton 2019). Given the vital role that Indigenous Peoples play in addressing the climate crisis, along with their unique susceptibility to its impacts (Ford et al. 2020), it is crucial to examine if NDCs recognise Indigenous Peoples. Previous research has explored the first round of NDC submissions (2016–2019), demonstrating that the consideration of Indigenous Peoples was marginal at best (Facilitative Working Group 2021a; Shea and Thornton 2019). Efforts have also been made to analyse this process from a regional perspective (Bijoy et al. 2022; Carmona et al. 2023). The UNFCCC, based on the advocacy by Indigenous Peoples and the direction from the Facilitative Working Group of the Local Communities and Indigenous Peoples Platform, has also begun paying more attention to this consideration, reporting in 2022 that 30% of the latest NDCs submitted by countries reference Indigenous Peoples but provide no detail on the type or depth of such consideration.^1^

Drawing from a collaboration between Indigenous and non-Indigenous scholars,^2^ we expand these efforts by critically analysing the first (2016–2019)^3^ and second (2020–2022) iterations of the NDCs, observing if/how this recognition has changed over time. First, we identify specific references related to Indigenous Peoples. Then, we assess whether these references promote or limit Indigenous Peoples’ sustainable self-determination. The level and quality of engagement these references promote are assessed through an analytical framework that draws on the dimensions of sustainable self-determination proposed by Reed et al. (2022). Combined, this analysis sheds light on whether and how States recognise Indigenous Peoples in the context of climate change, makes recommendations for the international community to facilitate their participation in climate governance better, and provides input to the Global Stocktake.

## Materials and methods

### Nationally Determined Contributions and Indigenous Peoples

Before 2015, Parties communicated their voluntary pledges to reduce greenhouse gas emissions through Intended Nationally Determined Contributions (INDC). Following the ratification of the Paris Agreement, all Parties were required to submit an NDC to the UNFCCC, outlining their strategy to reduce greenhouse gas emissions in line with the Paris target—to limit global warming to below 2, and preferably 1.5, degrees Celsius compared to pre-industrial levels—and adaptation targets. Regarding Indigenous Peoples, Parties are requested to provide information on planning and, if available, implementation plans in their NDCs, including ‘Domestic institutional arrangements, public participation and engagement with local communities and [I]ndigenous [P]eoples.’^4^ Most Parties submitted their first NDC between 2016 and 2019.

NDCs shifted the locus of control back to individual Parties (Pauw and Klein 2020). The voluntary approach allows NDCs to adjust to uncertainty and has been associated with increased ambition and credibility (Victor et al. 2022). Nevertheless, it has also raised concerns about their ability to produce concrete results, amongst others, because the proceeding allows for different interpretations (Geden 2016; Pauw and Klein 2020). For example, Parties that included targets up to 2025 in their first NDC were required to ‘communicate’ a new NDC by 2020, and Parties with targets up to 2030 or later—representing the majority of countries—were required to ‘update’ their NDCs by 2020. In either case, there is no agreed-upon definition for ‘communicate’ and ‘update’, enabling Parties to interpret and communicate them according to their preference (Pauw and Klein 2020). Whilst some Parties have made minor changes and adjustments alongside a report outlining their achievements, most countries have submitted an updated or, in some circumstances, an ‘enhanced’ version that represents a significant improvement compared to their first NDC. For our study, we refer to Intended and First NDC as ‘first submissions’ (*n* = 165), whilst we combine Updated, Enhanced and Second NDCs as ‘second submissions’ (*n* = 130).

The identification of countries with Indigenous Peoples is not straightforward. Indigenous Peoples are estimated to inhabit 90 countries, only 58 of which have generated information that allows official figures (ILO 2020). As a result, there is no official information to identify which countries have Indigenous Peoples, nor is there an official process to recognise Indigenous Peoples’ territories and nationalities that transcend national borders. Because of the above, we considered as cases for analysis all Parties that have submitted NDCs between 2016 and May 2022 (recuperated from https://unfccc.int/NDCREG).^5^

### Methods

The research was conducted through a summative and directed content analysis (Hsieh and Shanoon 2005), a method that assumes documents contain latent meanings, which we can access through how they present or omit specific issues (Berelson 1952). Beyond setting goals, NDCs are contested, negotiated, and ongoing discursive documents (Mills-Novoa and Liverman 2019). They identify problems and legitimise the knowledge, actions and actors in charge of climate action. Specifically, the mentions and omissions of content related^6^ to Indigenous Peoples in the NDCs give us insight into how states consider them in climate governance.

Summative content analysis identifies and quantifies keywords and content in text to understand its contextual use (Hsieh and Shanoon 2005). We coded paragraphs in all the NDCs searching for specific key terms (Table 1). Four authors—RC, FC, KP, and RY—conducted the coding and held weekly meetings for 3 months to discuss the results, avoid bias, and unify criteria. Paragraphs in NDCs that refer expressly to Indigenous Peoples at least once were selected for analysis. For example, references to ‘local community’ or ‘traditional knowledge’ were excluded if the NDC did not directly reference Indigenous Peoples.

**Table 1:** Codes used to identify references to Indigenous Peoples in the NDCs

Coding terms	‘Indigenous, traditional, people(s), community, local, ethnic, ethnicity, native, first nation, aboriginal, autochthonous, Indian, tribal, tribe, original, ancestral, pastoralists, pastoralism, nomadic, forest dweller, forest people, customary, worldview, cosmovision, knowledge [related to Indigenous knowledge systems], ways of knowing, consent [related to Free Prior and Informed Consent (FPIC)].’

Selected paragraphs were reviewed in-depth through a directed content analysis and were organised through categories (Table 2) previously defined based on the four-pronged framework of sustainable self-determination proposed by Reed et al. (2022), to which we added a fifth category. This framework addresses the main five claims that Indigenous Peoples have raised in the climate debates under the UNFCCC in order to strengthen their right to self-determination: (1) the poor implementation of the UNDRIP; (2) the impacts of territorial conflicts and the reproduction of colonialism through certain climate-related policies; (3) the undervaluing of Indigenous Peoples' knowledge systems; (4) the restrictions on participation in national and international spheres; and (5) the insistence on positioning Indigenous Peoples as vulnerable and therefore victims rather than agents in the face of climate change.

**Table 2:**
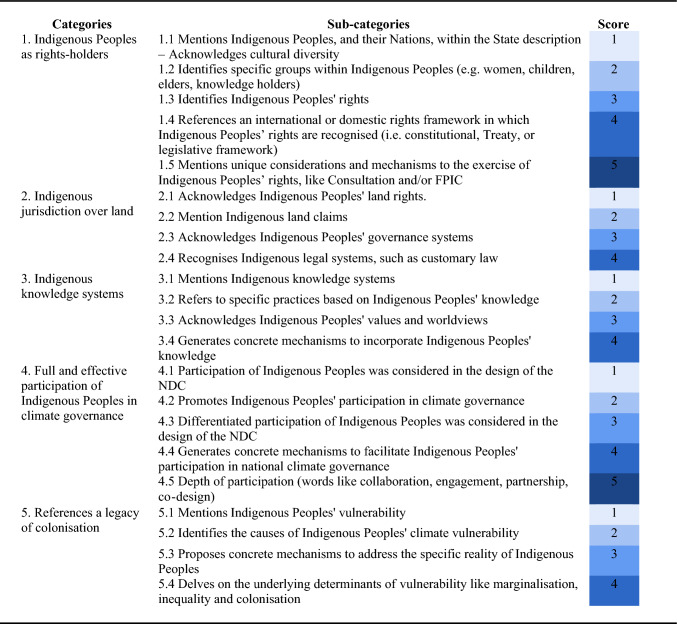
Assessment framework for the level and quality of Indigenous Peoples' engagement promoted by the NDCs (see supplementary material (Table S1) for a description of the scores)

Accordingly, we identified how the NDCs recognise (1) Indigenous Peoples as rights-holders; (2) Indigenous jurisdiction over land; (3) Indigenous knowledge systems; (4) the full and effective participation of Indigenous Peoples in climate governance; and (5) the legacy of colonisation. Each macro-category is broken down into 4 or 5 sub-categories associated with a score to assess the level and quality of recognition—between 1 and 5. These sub-categories, in contrast to the macro-categories, are the result of a dialogue between theory and content analysis of the selected paragraphs. The lowest scores (1 and 2) were associated with more superficial levels of recognition—i.e. a broader mention, identification or recognition of Indigenous Peoples without promoting a rights-based implementation of this recognition—whilst the highest scores (3, 4 and 5) correspond to stronger levels of recognition—i.e. the NDC promotes meaningful involvement and also explicitly commits to concrete mechanisms for its operationalisation. To assess inter-coder reliability we established a coding manual, which included the code name and description. This manual was developed after the first reading of the NDCs and the search of the keywords. Furthermore, we implemented a cross-coding strategy, where each coder author reviewed at least one other coder's coding to ensure a unified criteria.

We organised the NDCs into two categories: the first and second rounds of submissions. The scores allowed us to identify global trends and patterns concerning the levels of recognition set out in the NDCs. Ordinality should be understood in a qualitative and non-standardised manner; macro-categories are not comparable, so each sub-category has significance in its own context. Sub-categories aim to illustrate trends and differences between NDCs regarding recognition. Nevertheless, the order does not replace a more complex understanding of the recognition. The criterion that unifies the order of the categories is the reliability of the recognition, i.e. whether this recognition is expressed only in the discourse or whether it is possible to identify concrete mechanisms that guarantee it. The ranking was established based on the following questions: Does the NDC mention that it recognises Indigenous Peoples' rights? Does the NDC recognise these rights within a general framework of rights or as specific rights? If so, does the NDC mention practices that will guarantee the exercise of these rights, and does the NDC establish or create institutional mechanisms for such purposes? Finally, does the NDC consider collaboration within these mechanisms?

We identified progress concerning the number and depth of mentions of Indigenous Peoples. Then, we looked specifically at how the NDCs recognise Indigenous Peoples' rights and jurisdiction over land. Subsequently, we identified how NDCs recognise Indigenous Peoples' contributions to climate action, whether they consider and promote their knowledge and encourage participation. Finally, we examined how NDCs recognise and address the legacy of colonialism regarding climate change.

## Results

**Fig. 1:**
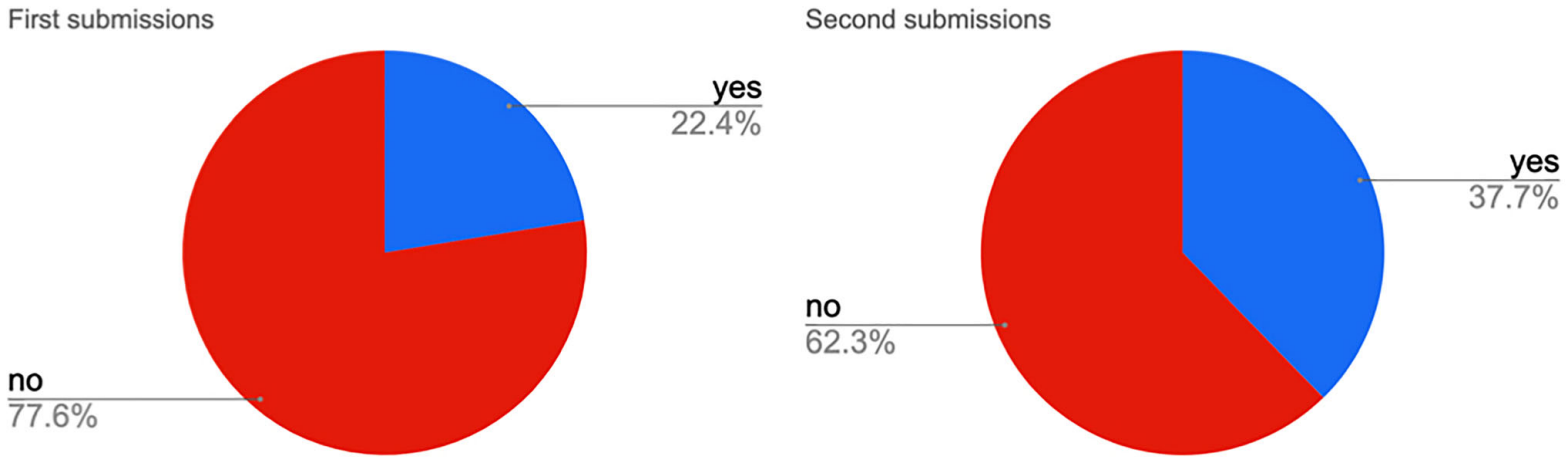
NDCs with references related to Indigenous Peoples

References related to Indigenous Peoples in NDCs are increasing (Fig. 1, see also supplementary material Table S2). Out of 295 documents, we identified 86 with such references: 37 from the first round of submissions (21% of the 165 NDCs) and 49 from the second (37% of 130 NDCs).

**Fig. 2:**
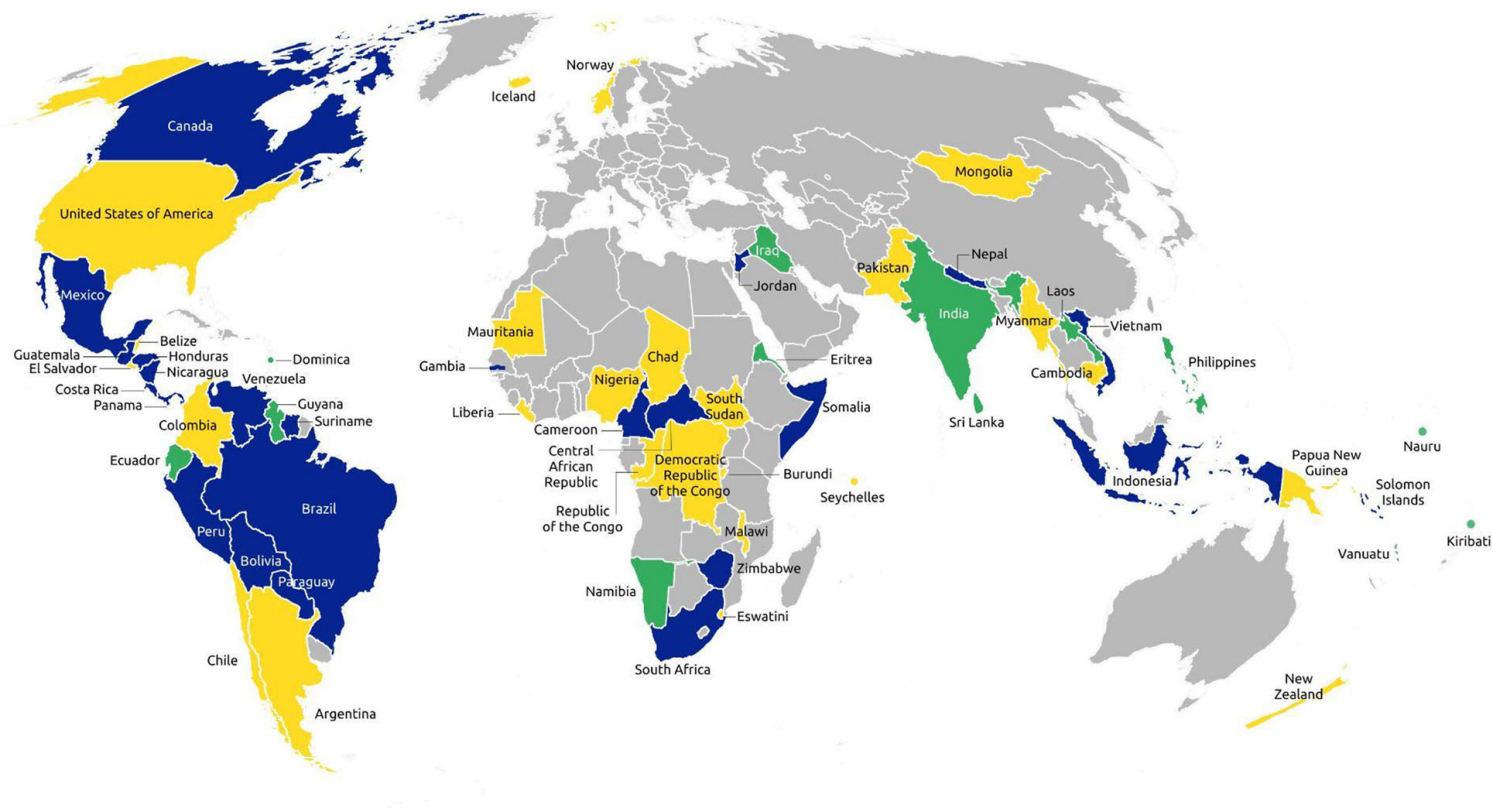
NDCs with references related to Indigenous Peoples in the first and second submissions. Green represents Parties that only made a first submission which included references related to Indigenous Peoples. Yellow represents Parties that made a second submission which included references related to Indigenous Peoples. Blue represents Parties that made a first and second submission which both included references related to Indigenous Peoples. Map elaborated by Robert Petitpas

Amongst the 37 Parties that refer to Indigenous Peoples in their first submission, 26 (70%) submitted a second or updated NDC. Of these, the majority (92%) include such references again, except Lao People's Democratic Republic and Sri Lanka—Parties that only referred to Indigenous knowledge in their first NDCs. Of the 49 NDCs with references in the second submission, 25 (51%) include references related to Indigenous Peoples for the first time (Fig. 2). This is worth noting as it indicates a trend of increased awareness and willingness of governments towards Indigenous Peoples in climate governance.

**Fig. 3:**
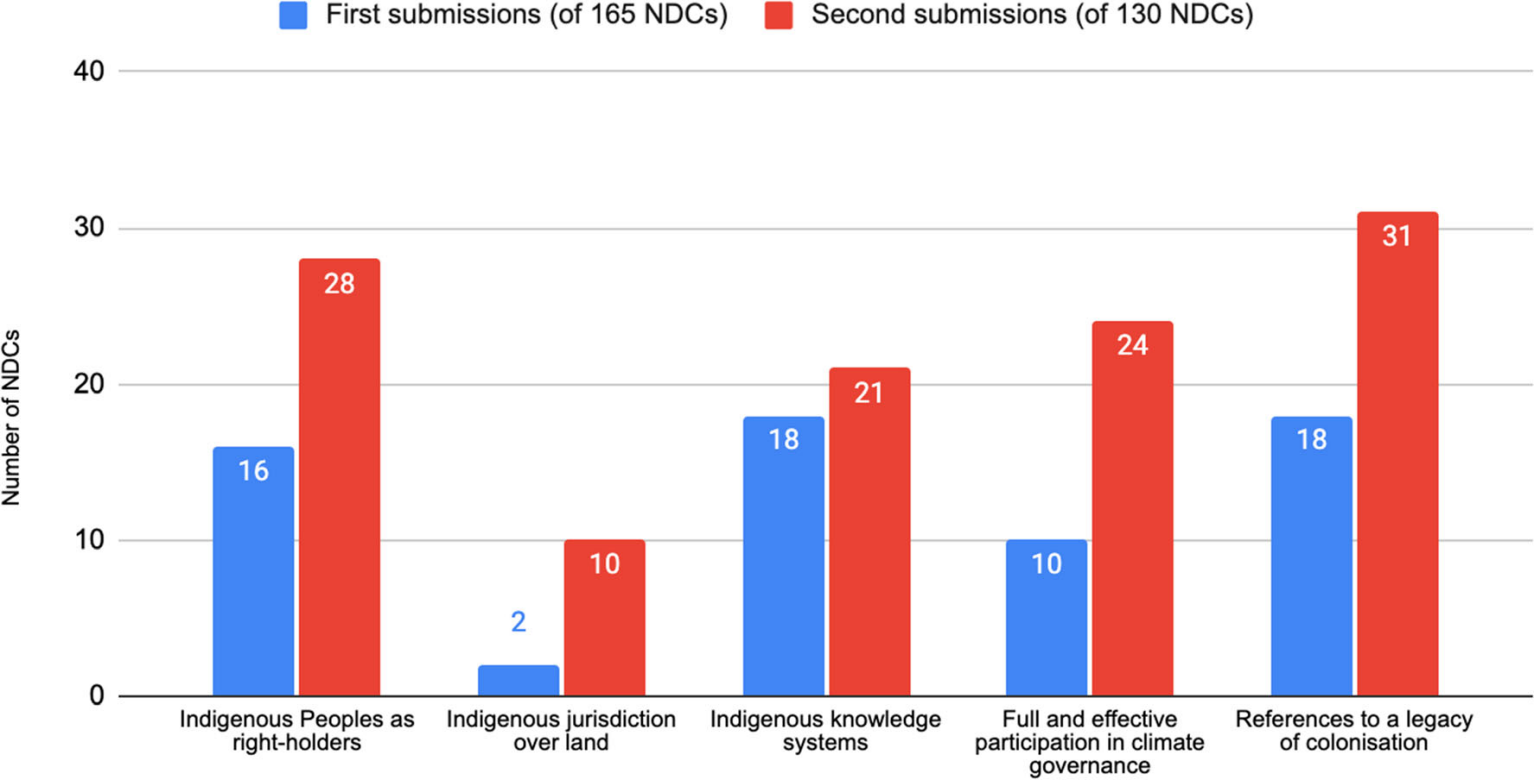
References by category in first and second submissions

In the first submissions, the most common reference was a tie between the role of Indigenous knowledge within climate action (*n* = 18) and the impacts of colonialism (*n* = 18). All reference categories increased in the second submissions (Fig. 3); however, the most significant increase was seen in the promotion of ‘participation’ (*n* = 24), where the number of NDCs nearly doubled. Another significant increase was in the number of references to ‘jurisdiction’—jumping from 2 NDCs in the first round to 14 in the second. NDCs mentioning 'Indigenous knowledge' in the second round of submissions only increased by 3, representing the lowest increase across all five categories. The most common reference in the second submissions relates to the 'impacts of colonialism' (*n* = 31). NDCs with mentions in only one category most often refer to Indigenous knowledge (9 NDCs in the first round and 8 in the second), and recognition of rights (5 NDCs in the first round and 4 in the second).

The combination of references, although present in both rounds of submissions, is higher in the second round. In the former, it is more common for NDCs to have references only in one or two categories, whilst in the latter, it is more common for references in NDCs to cover 3 and 4 categories. Because the recognition of knowledge and the recognition of the legacy of colonialism—i.e. vulnerability—are the most common references, these categories tend to be presented alongside each other. In the first round of submissions, the combination of these categories is the most common (7 NDCs). In the case of the second submissions, the most present combination is the one that links the recognition of the impact of colonialism with the recognition of Indigenous Peoples’ rights (21 NDCs). Another important combination in both rounds is the recognition of the legacy of colonialism together with the promotion of participation (6 NDCs in the first round and 20 in the second). The combination of recognition of rights with promotion of participation also stands out (5 NDCs in the first round and 17 in the second). No submissions in the first round have references across all five categories, but the Democratic Republic of the Congo, Costa Rica, and Canada do in the second round (see supplementary material Table S2).

From the above, we can conclude not only do more second round NDCs include references related to Indigenous Peoples (16%), indeed the quantity of references on average within each NDC has also increased. This is significant. We will now turn towards an analysis of references in each of the five categories.

### Recognition of Indigenous Peoples’ rights and jurisdiction over land

#### Indigenous Peoples as right holders

In both rounds of submissions we found a direct correlation between levels of recognition of rights and promotion of participation, as well as a correlation between the levels of recognition of rights and recognition of Indigenous knowledge. In the second round of submissions, we found that recognition of rights is also associated with greater recognition of the legacy of colonialism.

In the first round of submissions, 9 NDCs (5% of 165) acknowledged the existence of Indigenous Peoples within their national territory, whilst in the second round of submissions, this number grew to 15 (12% of 130). Only Guatemala refers to Indigenous women in the first round of submissions, whilst Panama and Vietnam reference them in the second round. In both cases, the reference relates to the specific vulnerabilities that Indigenous women face.

**Fig. 4:**
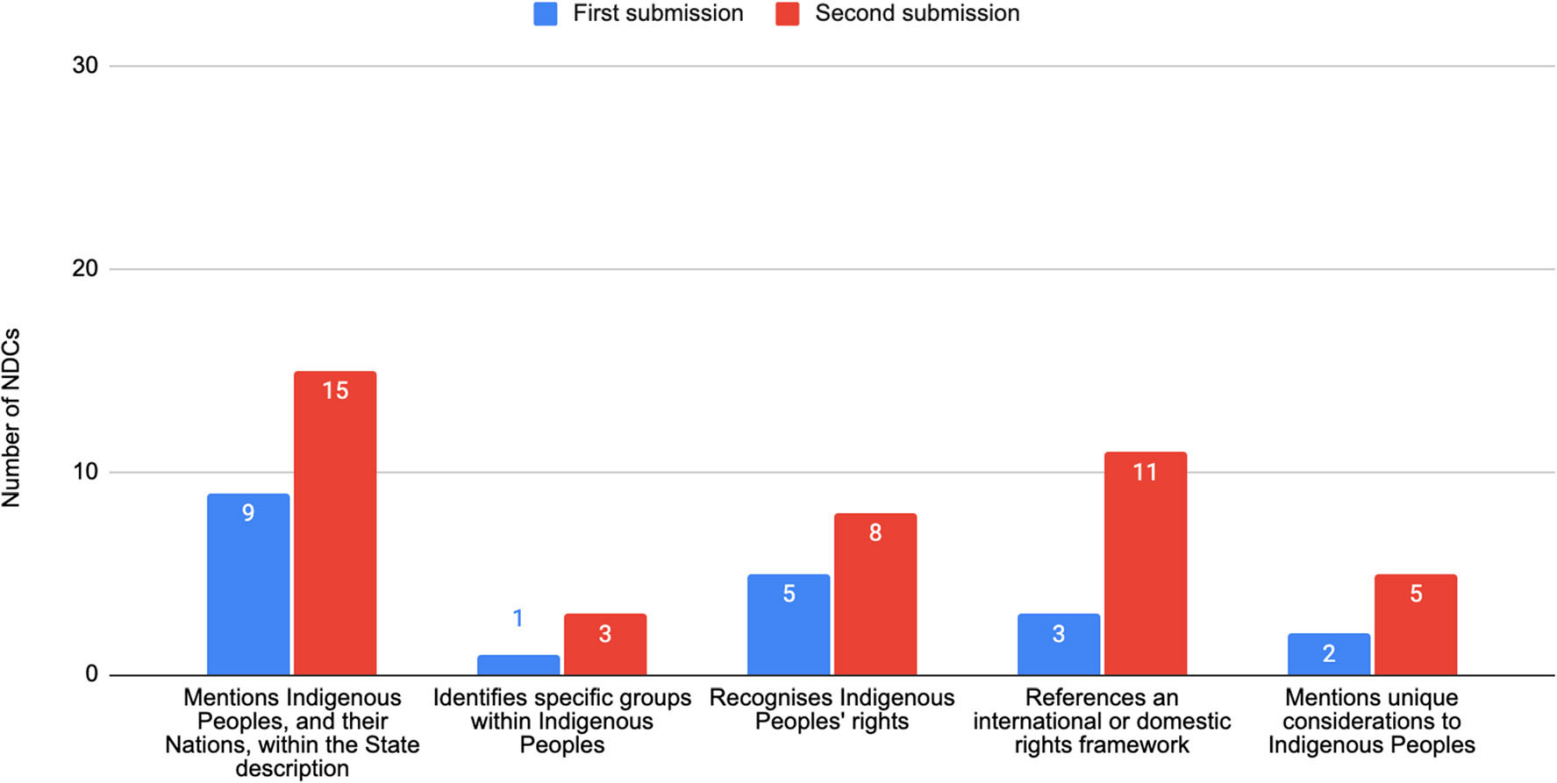
NDCs recognising Indigenous Peoples as rights-holders

In the second round of submissions, 8 (5%) NDCs explicitly refer to Indigenous Peoples' rights, 3 mention an international or domestic rights framework, and only 2 reference unique considerations (Fig. 4). Within the second round of submissions, the NDCs that refer to Indigenous Peoples' rights increased to 14% of the total number of submitted NDCs. In particular, 8 NDCs expressly recognise Indigenous Peoples' rights, and 11 refer to specific international or domestic rights frameworks, compared to 5 and 3 NDCs in the first round. There are 4 NDCs that refer to both issues, such as Aotearoa-New Zealand, which states that it respects the interests and rights of Indigenous Peoples and will consider the Treaty of Waitangi. There are only 5 NDCs that mention unique considerations: Nepal and El Salvador reference FPIC; Costa Rica and Panama refer to consultation; and Canada refers to self-determination. For example, El Salvador’s second NDC mentions that FPIC is implemented ‘for obtaining funds and facilitating mechanisms from international cooperation, as well as for the benefit of the population in the fulfilment of their rights.’

#### Indigenous jurisdiction over land

**Fig. 5:**
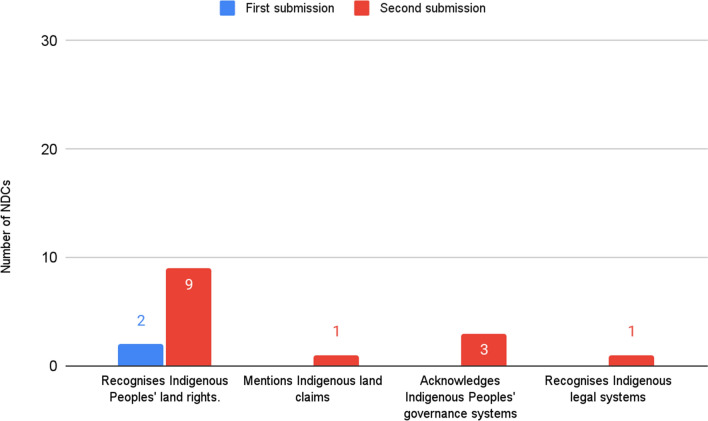
NDCs with references to Indigenous jurisdiction over land

Recognition of Indigenous Peoples' jurisdiction is marginal in both rounds, although the number of NDCs referencing Indigenous Peoples' land rights increased in the second (Fig. 5). In both rounds, most references acknowledge the specific role of Indigenous territories within conservation efforts.

In the first round of submissions, only 2 NDCs (1% of 165), those of Brazil and Guyana, loosely mention Indigenous land rights, whilst in the second round, we found 10 NDCs (8% of 130). For instance, Guyana’s first NDC states that ‘Indigenous people own and manage some 14% of Guyana’s lands. (…) Indigenous peoples themselves, through the full application of the FPIC process, in keeping with the stated policy in the LCDS [Low Carbon Development Strategy], will decide whether or not to include their titled lands as part of Guyana’s REDD + programme.’

References to jurisdiction over land are expanded in the second round of submissions, where for instance, Nicaragua details its specific legal framework protecting Indigenous territories and land rights. Other references in the second round of submissions include Indigenous Peoples' governance systems (*n* = 3), Indigenous land claims (*n* = 1) and Indigenous legal systems and customary laws (*n* = 1).

It is worth mentioning that all submissions that do not recognise jurisdiction prioritise a vulnerability-based approach when referring to Indigenous Peoples.

### Recognition of Indigenous Peoples’ contributions to climate action

Recognition of Indigenous Peoples' contributions—expressed through the recognition of knowledge and the promotion of participation—is increasing and it is greatly related to the recognition of Indigenous Peoples’ rights. Whilst NDCs express recognition of Indigenous knowledge in both rounds, we can observe a greater promotion of participation in the second round.

#### Recognition and promotion of Indigenous Peoples’ knowledge

**Fig. 6:**
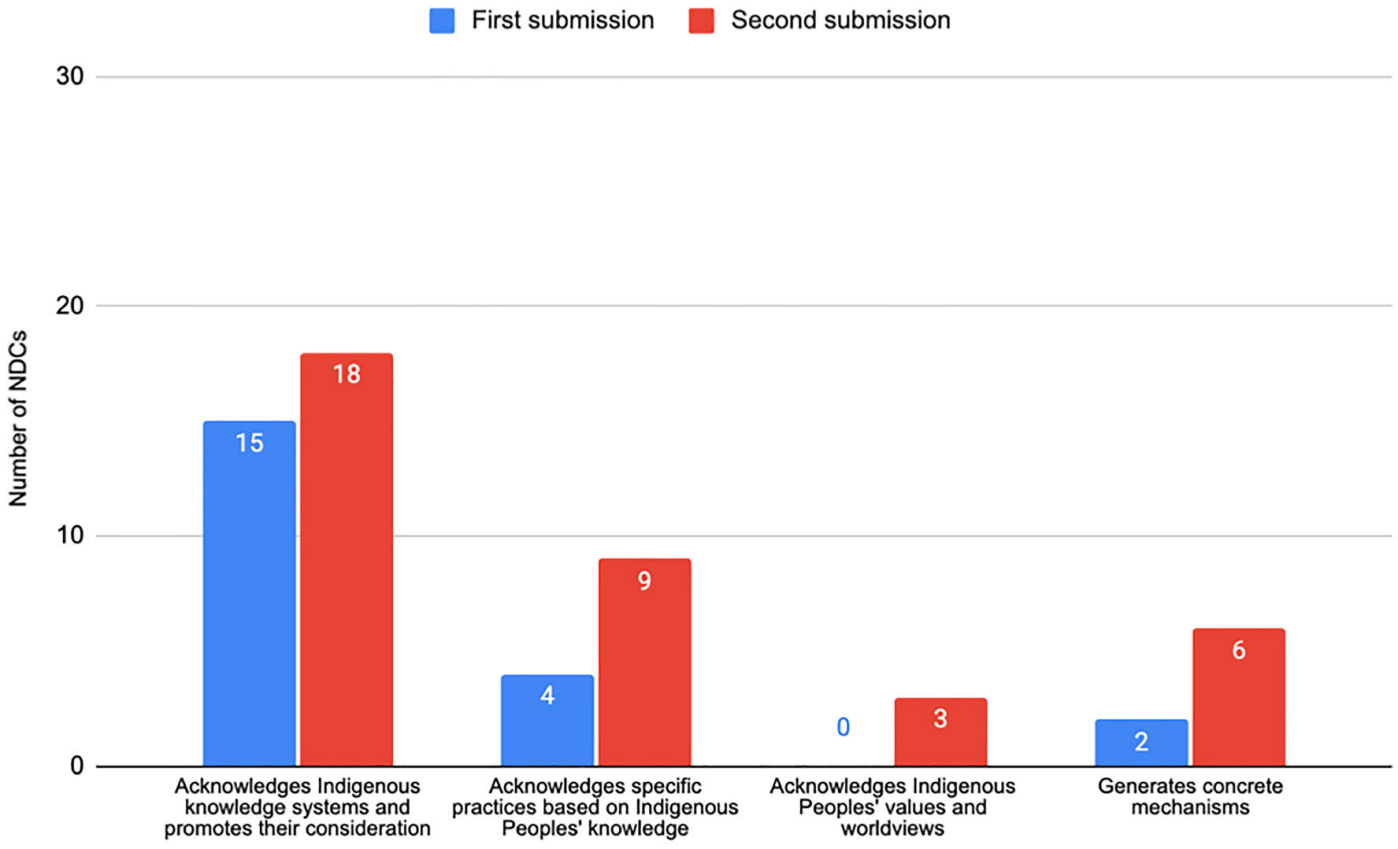
NDCs recognising Indigenous knowledge systems

References to Indigenous knowledge are the highest across all five categories in the first round of submissions (see Fig. 6, 11%). This recognition, however, is superficial as only 4 NDCs describe specific practices used by Indigenous Peoples, and only 2 NDCs promote mechanisms to integrate this knowledge: Guyana and Venezuela. No NDCs recognise Indigenous Peoples' visions and values.

Although the increase in references to Indigenous knowledge in second submissions is low (16% of 130), these references are more specific. Of these submissions, 18 NDCs (14%) directly promote consideration of Indigenous knowledge. There are also specific references to the practices used by Indigenous Peoples (9 NDCs) and to Indigenous Peoples' values and visions (3 NDCs). For example, in its updated NDC, Paraguay notes that it embraces Indigenous Peoples' cosmovision for territorial and centralised climate action. There is also an increase in NDCs referring to concrete mechanisms for incorporating Indigenous Peoples' knowledge (6 NDCs compared to 2 in the first round). For example Aotearoa-New Zealand refers to Vision Mātauranga, ‘a government policy that aims to unlock the science and innovation potential of Māori knowledge, resources and people for the environmental, economic, social and cultural benefit of New Zealand.’

#### Promotion of full and effective participation

**Fig. 7:**
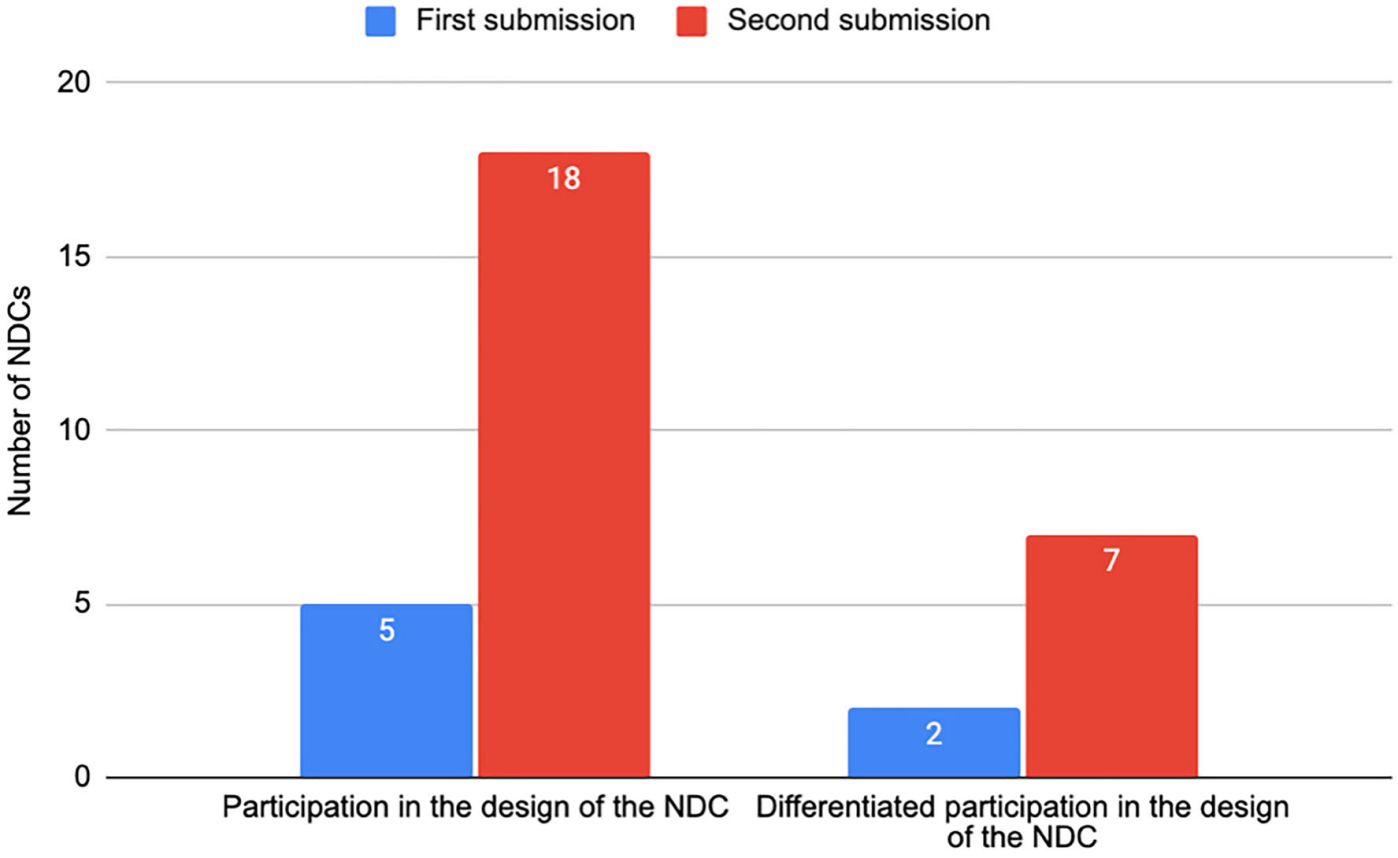
Indigenous Peoples’ participation in NDCs preparation

Five NDCs (3% of 165) in the first round of submissions considered the participation of Indigenous Peoples during the preparation (Fig. 7). Of these, only two countries conducted consultative processes that considered Indigenous Peoples as distinct actors in the climate policy discussion: Panama held public hearings with representation from Indigenous Peoples' representative institutions, and Guyana presented the draft of the NDC at a meeting targeted at representatives of Indigenous organisations and communities.

In the second round of submissions, NDCs with references to the participation of Indigenous Peoples during preparation increased from 5 to 18 (14% of 130). Of these, 7 explain the specific and differentiated processes that Parties took to include Indigenous participation: 5 described a process whereby Indigenous Peoples and their representative institutions participated in sessions that included stakeholders and other non-Indigenous organisations; and only 2 described a process whereby Indigenous Peoples were convened in an Indigenous-specific process.

**Fig. 8:**
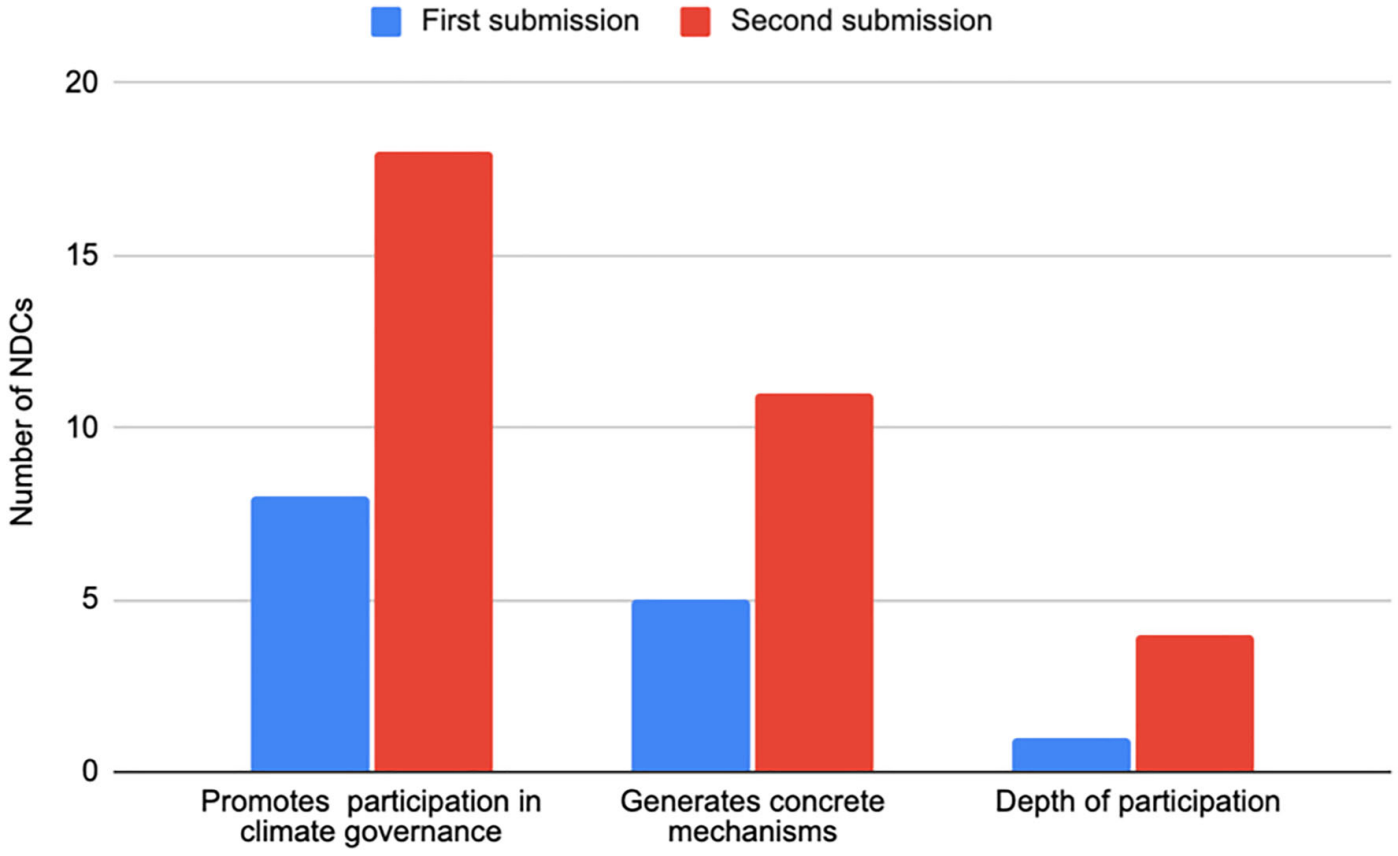
NDCs promoting Indigenous Peoples’ participation in implementation plans

References to Indigenous Peoples' participation in implementation plans grew significantly between the first and second rounds of submissions (Fig. 8). In the first round, 8 NDCs (5% of 165) referred to promoting participation in climate governance, and 5 (3%) described concrete mechanisms for supporting this participation. Indonesia, for example, referenced Indigenous Peoples' participation in conservation measures, whereas Peru encouraged the participation of Indigenous organisations in climate action. Canada is the only Party describing the depth of this participation as ‘meaningful engagement’ (p. 7).

In the second round of submissions, 18 NDCs (14% of 130) encouraged the participation of Indigenous Peoples within climate governance, and 11 (8%) mentioned concrete mechanisms to facilitate it. Four NDCs described the depth of participation: the Democratic Republic of the Congo, Myanmar, and Nepal mention the partnership with Indigenous Peoples, whilst Canada refers to Indigenous Peoples' leadership. Specifically, Canadas’ updated NDC mentions that ‘the Government of Canada has been and will continue to partner with First Nations, Inuit, and the Métis Nation to position Indigenous climate leadership as a cornerstone of Canada’s Strengthened Climate Plan and ensure that federal initiatives support Indigenous Peoples’ climate priorities and ambitions.’

### Recognising the legacy of colonialism

**Fig. 9:**
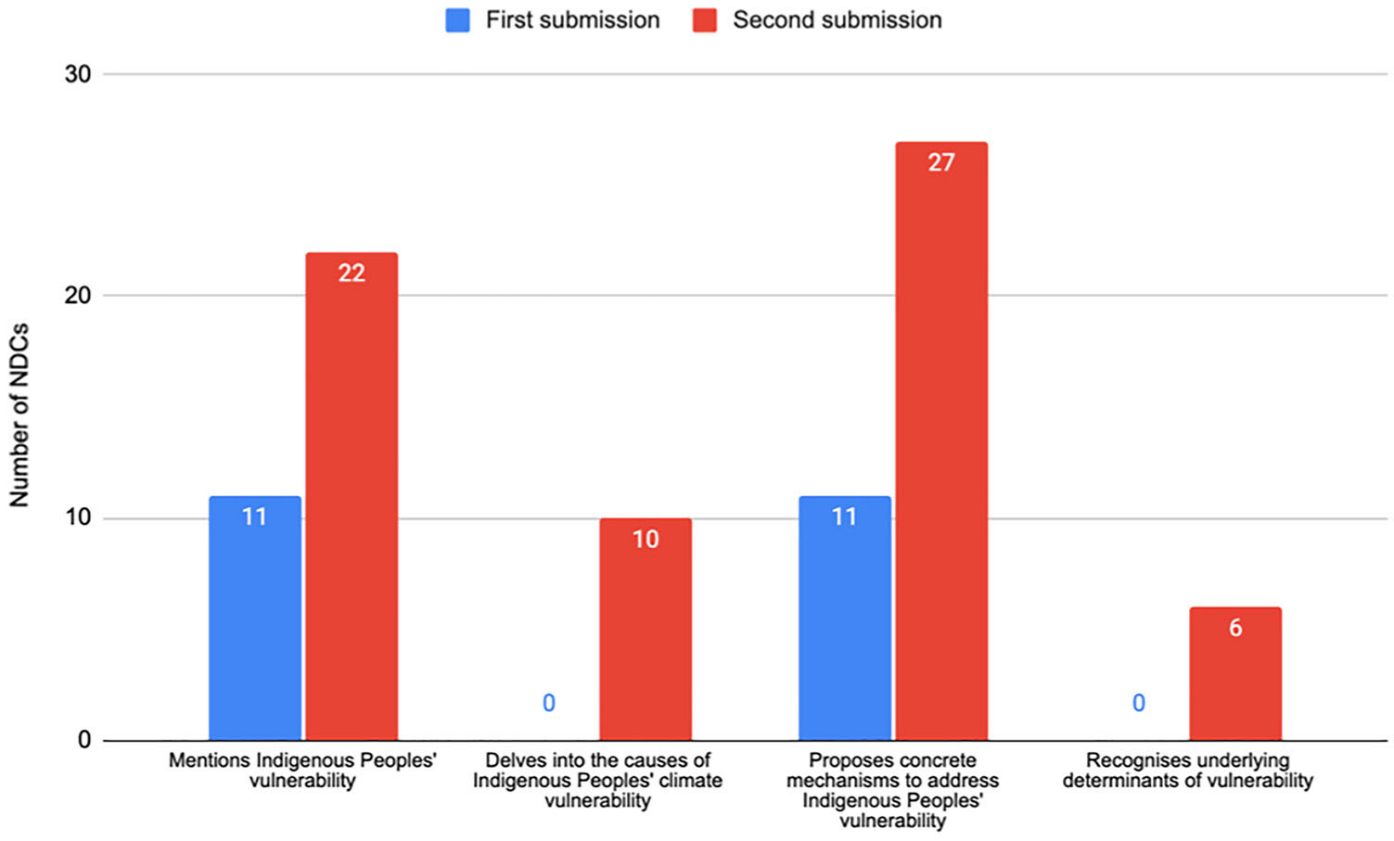
NDCs recognising the legacy of colonialism

There are numerous indirect references to colonialism in both rounds of NDCs, often captured within the description of the climate change impacts faced by Indigenous Peoples (Fig. 9). Nevertheless, Canada and Bolivia are the only two that directly acknowledge the impact of colonisation.

In the first round of submissions, 11 NDCs mention that Indigenous Peoples are amongst the most affected groups. For instance, Vanuatu describes how climate change will affect all areas of the Ni-Vanuatu People. Despite this, no NDCs elaborate on the causes of this vulnerability beyond their dependence on and relationship to the land, water, and territories. Furthermore, 11 NDCs (7% of 165) refer to concrete climate action being implemented within Indigenous territories, though often not led by Indigenous Peoples. Finally, amongst the first submissions, no NDCs directly referenced the underlying determinants of vulnerability, such as marginalisation, inequality, and colonisation.

NDCs explicitly mentioning Indigenous Peoples' vulnerability to climate change doubled in the second round of submissions, accounting for 17% (22 out of 130). This round shows a considerable increase in the references to concrete measures that help respond to the vulnerability of Indigenous Peoples, amounting to 27 NDCs, or 21% of the total. However, most of these measures do not refer to concrete participation mechanisms. Amongst the NDCs that do make mechanisms explicit, El Salvador's refers to the creation of methodologies—including FPIC—to ensure the appropriate participation of Indigenous Peoples. There is also an increase in references to the underlying causes of Indigenous Peoples’ climate vulnerability (6 or 5% of 130). For example, Canada’s updated NDC mentions that the ‘compounding and interconnected impacts of climate change, lower socio-economic outcomes, colonial legacies, and disparities in access to clean technologies have had and continue to have an important impact on Indigenous Peoples’ wellbeing.’

## Discussion

The references to Indigenous Peoples in NDCs are increasing in both number and substance. Countries are recognising more specific elements and it is therefore more common for second submissions to have references in more than one category. These increases can be attributed to the significant advocacy of Indigenous Peoples at both the national (Reed et al. 2021) and international scale, including the operationalisation of the Local Communities and Indigenous Peoples Platform under the UNFCCC (Belfer et al. 2019; Shawoo and Thornton 2019). This increase aligns with recent COPs decisions, such as the Glasgow Climate Pact that ‘*Emphasizes* the important role of indigenous peoples’ and local communities’ culture and knowledge in effective action on climate change and *urges* Parties to actively involve indigenous peoples and local communities in designing and implementing climate action’ (Decision 1/CP. 26 par. 66, italics in the original). However, this is not enough to support Indigenous Peoples' meaningful inclusion. To contribute to this process, we return to our five-pronged framework of sustainable self-determination and discuss their implication for improving considerations of Indigenous Peoples within NDCs.

### Indigenous Peoples as rights-holders

Behind references to colonialism in 49 NDCs, references to Indigenous Peoples as rights-holders are the second most common across the first and second submissions. The second round of submissions saw an increase in the number of references to Indigenous Peoples as rights-holders, reflecting a growing engagement with the Paris Agreement's rights-protections. It was common for submissions to reference Indigenous Peoples and their Nations, followed by references to Indigenous Peoples' rights and international or domestic rights frameworks.

In both rounds, high levels of recognition of rights tend to be associated with greater recognition of Indigenous Peoples' contributions—through higher promotion of participation and recognition of Indigenous knowledge. Nevertheless, mainstreaming a rights-based approach remains marginal in the broader context of NDC implementation, as 17 mention the Indigenous population without mentioning their rights, and 20 expressly recognise them—either by directly declaring it or referring to an instrument that protects them. For instance, Guyana and Costa Rica refer to FPIC in the context of Reducing Emissions from Deforestation and Forest Degradation (REDD +) projects.

This gap can be attributed to the continued reluctance of states to acknowledge and uphold the rights of Indigenous Peoples (IACHR and IWGIA 2021), despite this recognition being a prerequisite for climate action, especially when it is implemented in their territories. Furthermore, many Parties that refer to Indigenous Peoples in their NDCs face conflicts over the demarcation and administration of territory that hinder collaboration (Townsend et al. 2020). Amongst these conflicts, we can highlight the appropriation of Indigenous land, waters, and territories and the imposition of projects that constrain Indigenous Peoples' ways of life and right to their own development in the name of climate action (Loaiza et al. 2017; Ulloa 2017). For example, in the Andes (Chile, Argentina, Bolivia), most lithium concessions overlap with Indigenous territories facing water scarcity, pollution and biodiversity loss (Voskoboynik and Andreucci 2022). In Norway, the Saami People are demanding the removal of wind farms installed in their territory without their consent.^7^

If NDCs do not consider how states address these conflicts, they are likely to reinforce colonial dynamics that produce vulnerability (Ford et al. 2020) and risk the effectiveness and coherence of climate policy (Carmona 2023).

### Indigenous jurisdiction over land

Whilst there is an urgent need for climate policy to respect the rights of Indigenous Peoples to their territories (Carmona et al. 2022), Indigenous jurisdiction over land received the least amount of references across the five categories. Canada is the only country that explicitly references Indigenous land claims and customary laws; however, these references are found in the annex prepared by the national Indigenous organisations, Assembly of First Nations, Inuit Tapariit Kanatami, and Metis National Council. Three NDCs—Canada, Norway, and the United States of America—reference Indigenous governance, referring to First Nations, the role of the Sámediggi, and the authority of Tribal Governments, respectively. In both rounds, references to Indigenous jurisdiction correlate with more recognition of rights, including the right to participation.

These findings are not entirely surprising; many Parties fear the advancement of Indigenous Peoples' jurisdiction and their self-determination threaten a loss of sovereignty or territorial integrity (Lightfoot and MacDonald 2017). These fears are contrary, however, to the growing amount of evidence that acknowledges safeguarding the rights and jurisdiction of Indigenous Peoples is linked to more effective mitigation and adaptation policies (Farbotko and McMichael 2019; RRI 2021). They also overlook the claims of different Indigenous scholars who have pointed out that recognising Indigenous legal systems is critical to supporting Indigenous climate leadership (Reed et al. 2022). Canada’s updated NDC is one of the few to recognise this opportunity by calling for ‘immediate, transformative action built on the recognition, respect, and safeguarding of First Nations governance, rights, and jurisdiction, advancing the Government of Canada’s commitment to positioning First Nations climate leadership as a cornerstone of its climate efforts’ (p. 40).

### Indigenous knowledge systems

The contributions of Indigenous knowledge systems to climate change and biodiversity solutions have been increasingly recognised at the local, national, and international scale (Garnett et al. 2018; Adade Williams et al. 2020). This recognition is reflected in the references to Indigenous knowledge within the NDCs, as they are the highest across all categories in the first round of submissions, with a small increase in the second round of submissions. Nevertheless, most references are acknowledgements of the existence of Indigenous knowledge rather than substantive recognition of specific practices and Indigenous Peoples’ values and worldviews, which are critical to responding to climate change (Orlove et al. 2022). Amongst the exceptions, Venezuela describes a path of action that seeks to rescue the ancestral knowledge of Indigenous Peoples for the development of sustainable technologies, and Guyana states that the ‘culture and traditions of Guyana’s [I]ndigenous [P]eoples are rooted in sustainable use of nature, evident in the forests and other natural ecosystems maintained through centuries on the lands they have customarily occupied and used’ (p.17).

The absence of engagement with Indigenous Peoples’ values and worldviews may perpetuate an understanding of the climate crisis as one exclusively related to the reduction of greenhouse gas emissions (Chakrabarty 2019). Instead, Indigenous knowledge keepers have called for a re-evaluation of the framings of climate change towards one focused on how human values have created a world of imbalance, as Dakota Knowledge Keeper Katherine Whitecloud says:

People don’t want to acknowledge the state of the Earth, where it’s at right now, because it’s a reflection of themselves. It’s a reflection of their homes, their personal space, where the spirit and the heart reside… And people don’t want to look at that (Cameron et al. 2021, p. 43).

Furthermore, there is little commitment to implementing concrete mechanisms to facilitate collaboration or co-production of knowledge. Instead, Parties focus on the unilateral communication of information *to* Indigenous Peoples. Guatemala and Costa Rica, for instance, commit to channelling information to Indigenous Peoples through Agroclimatic Technical Roundtables and platforms. Aotearoa-New Zealand commits to promoting Māori-focused research and supporting Māori to create their own transition strategy, based on Māori knowledge and responding to Māori’s specific priorities and needs. However, there is no explicit discussion of how this research would be mainstreamed or inform the organisation of their NDC. The only exception could be Canada’s updated NDCs, as it allowed Indigenous Peoples to draft their contributions—nevertheless in the form of an annex.

Comberti et al. (2019) push for more than just bridging or integrating knowledge systems, calling for the *demarginalisation* of Indigenous Peoples within the UNFCCC based on substantive and collaborative engagement. This aligns with other scholars, such as Reed et al. (2020), who call for the concept of *braiding* knowledge systems to understand the multiple ontological and epistemological foundations that enter into knowledge valuation and co-production in a process of mutual respect, kindness, and generosity. This type of engagement can have a positive impact on Indigenous Peoples’ rights because it allows for the inclusion of the various components of Indigenous Peoples’ knowledge systems (Orlove et al. 2022).

### The full and effective participation of Indigenous Peoples in climate governance

The political marginalisation of Indigenous Peoples is one of the leading causes of their vulnerability to climate change (Ford et al. 2020) and therefore urgently needs to be reversed.

Although the participation of Indigenous Peoples in the preparation of NDCs has increased, the existence of dedicated spaces for Indigenous Peoples to influence the process remains marginal (Shea and Thornton 2019). For instance, Argentina’s second NDC describes a roundtable process that included Indigenous participation, and Colombia’s updated NDC refers to dialogues with Indigenous Peoples. Nevertheless, few Parties provide enough detail to assess if this engagement was consistent with Indigenous Peoples’ protocols and systems of representation and governance. Canada’s updated NDC referred to collaborative relationships with the Assembly of First Nations, Inuit Tapiriit Kanatami and the Métis National Council, specifically through the establishment of ‘three distinctions-based senior bilateral tables based on the recognition of rights, respect, co-operation, and partnership’ (p. 16). Outside of this reference, none of the NDCs makes explicit how Indigenous Peoples’ contributions were integrated into the document. The absence of dedicated participatory processes can be attributed to the lack of collaborative spaces in national climate governance (Gustafsson and Schilling-Vacaflor 2022; Carmona 2023).

The lack of participation during the preparation is reflected in a low ambition to create spaces for meaningful engagement in the NDCs implementation plans. In most cases, the engagement of Indigenous Peoples is limited to specific actions in their territories that seek to address their vulnerability, restricting Indigenous Peoples’ ability to contribute to climate governance in a more integrated, sustained and proactive way and reproducing in many cases top-down approaches that reinforce colonialism (Whyte 2021). Few NDCs commit specific mechanisms for the engagement of Indigenous Peoples. For example, the Democratic Republic of the Congo’s updated NDC notes that its operationalisation will only be possible through an inclusive approach that incorporates Indigenous Peoples, directing the Ministry of Environment and Sustainable Development to collaborate with them. Similarly, Nepal’s second NDC refers to the development of specific programmes and dedicated resources to ensure the participation of Indigenous Peoples, as well as the creation of forest management committees composed of Indigenous Peoples’ representatives. The absence of concrete mechanisms is worrying since the NDCs are neither binding nor formally punitive and lack standardised design guidelines and mechanisms to verify compliance (Victor et al. 2022).

In addition to enjoying the right to participation, Indigenous Peoples have consistently shown their capacity to respond to climate change more sustainably and justly (Adade Williams et al. 2020; Schlingmann et al. 2021). In contrast, states’ efforts to address the climate crisis have so far proven to be widely insufficient, as we rapidly move beyond planetary boundaries (Steffen et al. 2018; Armstrong McKay et al. 2022). Moreover, locally implemented responses have often led to adverse effects, such as the violation of Indigenous Peoples’ rights (Loaiza et al. 2017) and maladaptation (IPCC 2022b). Such failures can be highly attributable to the absence of rights-based participatory mechanisms (Lawrence et al. 2022). Therefore, without better standards of participation, it is uncertain how NDCs will avoid the reproduction of these shortcomings.

### The legacy of colonisation

In its latest report, the IPCC (2022b) recognises that Indigenous Peoples’ vulnerability is essentially a legacy of colonialism which, in addition to excluding them from decision-making processes, currently limits their capacity to respond (Carmona et al. 2022). Recognition of this vulnerability is necessary to promote actions to reverse it, but it is not a neutral exercise. Identifying Indigenous Peoples as vulnerable often results in the imposition of measures that, by positioning them as mere recipients of climate policies, reinforce the same colonial dynamics that make them vulnerable (Callison 2021). In fact, in the first round, all NDCs identifying Indigenous Peoples as vulnerable and/or proposing measures in Indigenous territories without referring to the underlying determinants of this vulnerability do not refer to Indigenous jurisdiction. Amongst these, there is also low promotion of participation and low recognition of rights and knowledge. The trend is quite similar in the second round. In addition to detracting from the agency of Indigenous Peoples, this approach restricts attempts of decolonisation that seek to minimise the negative impacts of Indigenous Peoples’ structural marginalisation, such as maladaptation (Lawrence et al. 2022).

Few Parties elaborate on the specific causes and recognise the underlying determinants of vulnerability. For example, Vietnam’s updated NDC describes how Indigenous Peoples living in mountains are exposed to floods and storms. In describing these impacts, it also refers to non-economic losses—which the NDC recognises are more significant than economic losses—such as health impacts, those associated with relocation, loss of land due to erosion, loss of cultural heritage and local knowledge, and loss of biodiversity and ecosystem services. To address the direct causes of vulnerability, the states’ pledge in the second round of submissions measures that range from capacity building, as expressed by Cameroon and Mexico, to adaptation strategies, as noted by Vietnam, and ecosystem repair, described by Honduras and Nicaragua. Whilst all of these actions are relevant, it is worth mentioning that most of them do not refer to participation mechanisms. Canada is the only country that commits a specific budget for these measures, pledging support for Indigenous communities’ energy transition.

Fewer Parties, and only in the second round, recognise the underlying determinants of climate vulnerability. We found references to education access (Vietnam); marginalisation (Guatemala); poor participation in decision-making (Argentina and Bolivia); the impact of colonisation (Canada); and inequality and a structural system that creates vulnerability (Mexico). The annex produced by the Assembly of First Nations in Canada makes this reference explicit, stating that ‘it is clear that climate efforts must incorporate and address the systemic inequities and gaps that have resulted from the historical and ongoing impacts of colonisation, land dispossession, and assimilationist policies’ (p. 39).

## Conclusion

In this study, we provided a global overview of the incorporation of Indigenous Peoples within NDCs using a five-pronged sustainable self-determination framework. Based on this analysis, we can conclude that the NDCs with references related to Indigenous Peoples are increasing globally. We see the recognition of Indigenous Peoples’ rights augmenting and becoming more concrete through mechanisms for their implementation. However, this does not extend to the recognition and inclusion of Indigenous jurisdiction, which remains marginal and largely absent in NDCs. References to Indigenous knowledge systems are slowly increasing; however, questions remain regarding their sincerity and commitment to implementation. The same applies to references to the full and effective participation of Indigenous Peoples, especially concerning the involvement of Indigenous Peoples in the implementation of climate policy. Finally, the explicit recognition of the legacy of colonisation is rare; however, references to the vulnerabilities of Indigenous Peoples to climate change remain common.

Our findings illustrate, to a large extent, the outcomes of Indigenous Peoples’ international advocacy under the UNFCCC. Recognition is increasing in discourse and pledges; however, the demands of Indigenous Peoples to strengthen their rights have not yet been met. Parties are missing the opportunity to build trust with Indigenous Peoples and thereby enable the conditions that will allow them to contribute to climate governance. On the contrary, they continue reinforcing the political marginalisation that constraints Indigenous Peoples’ resilience and adaptation capacity.

Although this global analysis serves as input to the current Global Stocktake, we recognise limitations in focusing exclusively on the written content. References in NDCs are important because they allow Indigenous Peoples’ movements to use them to hold states to account and demand they honour the commitments made to Indigenous Peoples in implementation. Nevertheless, this is only the first step towards the full and effective implementation of Indigenous Peoples’ rights in climate governance and safeguarding in climate action. Complying with Indigenous Peoples’ rights is first and foremost a moral obligation. However, safeguarding these rights is essential to achieving any meaningful intervention’s climate objectives. If this does not happen, references to Indigenous Peoples in the NDCs risk being understood as containment of claims and a legitimisation mechanism before the international community—not an effort to give Indigenous Peoples the place they deserve in climate action.

The coherence of NDCs should be assessed through their implementation in each context. Future research must be conducted in dialogue with Indigenous Peoples and state representatives engaged in NDC preparation and implementation and analyse the diverse ways Indigenous Peoples are invited to and engaged in country-level climate governance.

To strengthen coherence, states should ensure that NDCs are consistent with the minimum standards established by the UNDRIP. Furthermore, national climate pledges should explicitly state how their implementation considers Indigenous Peoples’ land and water rights and respects Indigenous governance systems. This recognition requires a clarification of how Indigenous jurisdiction is integrated into climate policy and how conflicts that restrict Indigenous Peoples’ governance are taken into consideration.

The implementation of NDCs must be done in dialogue with Indigenous knowledge systems. But first, Parties need to remember that the Indigenous Peoples’ knowledge is the knowledge of the rights-holders (van Bavel et al 2022). Any engagement of these knowledge must occur in contexts that respect Indigenous Peoples’ rights, including sovereignty over the information they provide. Furthermore, NDCs should strengthen Indigenous-led research and clarify how collaboration is incorporated into implementation—considering all components of Indigenous knowledge systems, such as values, worldviews and protocols (Orlove et al. 2022). Strengthening Indigenous knowledge systems will also strengthen customary legal systems, facilitating better territory stewardship and more just climate action. NDCs must build capacity and secure financial support to increase the effective, respectful, equitable, consistent and ongoing engagement of Indigenous Peoples at the national and local levels—always respecting the collective rights of the Peoples, communities, and individuals involved.

Finally, any climate action, in addition to focusing on the future, must also take account of the past. Otherwise, we risk reproducing the same structures that created the problem in the first place. Parties must go beyond recognising Indigenous Peoples’ vulnerability and address its underlying determinants. NDCs must secure mechanisms to overcome the ongoing legacy of colonialism and commit financial and technical support for Indigenous-led projects. Together, these actions will contribute to strengthening the self-determination that Indigenous Peoples, as sovereign nations under international law, deserve at all levels of climate governance.

### Supplementary Information

Below is the link to the electronic supplementary material.

Supplementary file1 (PDF 114 kb)

## Footnotes

1 See https://unfccc.int/sites/default/files/resource/cma2022_04.pdf

2 The first output of this collaboration was a policy paper that presented part of the results of this research, available here https://www.iwgia.org/en/resources/publications/4943-recognition-indigenous-peoples-nationally-determined-contributions.html.

3 Some countries submitted their first NDC after this date but have been included in this round.

4 Decision 4/CMA.1 available at https://unfccc.int/sites/default/files/resource/cma2018_03a01E.pdf

5 All European Union countries submit the same NDC. We analysed the NDCs of 194 Parties: the NDCs of 193 countries plus that of the EU (which includes other 27 countries).

6 By “related” we mean not only direct references to Indigenous Peoples, but also broader references concerning them.

7 See https://www.globalissues.org/news/2023/03/09/33273
