# Supplementary material for: Indigenous Peoples’ rights in national climate governance: An analysis of Nationally Determined Contributions (NDCs)
Source: Ambio. 2023 Oct 11;53(1):138–55. doi: 10.1007/s13280-023-01922-4 (PMC10692065; doi:10.1007/s13280-023-01922-4)
Supplement: Supplementary file 1 — Supplementary file1 (PDF 114 kb) [file 13280_2023_1922_MOESM1_ESM.pdf]

Ambio

Electronic Supplementary Material
This supplementary material has not been peer reviewed.

Title: Indigenous Peoples' Rights in National Climate Governance: An Analysis of Nationally Determined Contributions (NDCs)

Authors: Rosario Carmona, Graeme Reed, James Ford, Stefan Thorsell, Rocío Yon, Francisca Carril, Kerrie Pickering.

Table S1. Assessment framework for the level and quality of Indigenous Peoples' engagement promoted by the NDCs with a description of the scores

Categories	Score	Description
1. Indigenous Peoples as rights-holders	1	Indigenous Peoples are mentioned in the general description of the state as part of cultural diversity
	2	NDC mentions specific groups within Indigenous Peoples, such as Indigenous women, Indigenous children and youth
	3	NDC identifies Indigenous Peoples as a specific group, with specific rights. Nevertheless, it does not mention specific instruments that guarantee the exercise of these rights
	4	NDC tresses that the rights of Indigenous Peoples are recognised in specific instruments of national legislation
	5	NDC refers to the mechanisms through which Indigenous Peoples exercise their specific rights, e.g. Indigenous Peoples' Prior Consultation and FPIC.
2. Indigenous jurisdiction over land	1	NDC identifies the existence of territories inhabited by Indigenous Peoples
	2	NDC mentions Indigenous Peoples' claims for restitution of their territory
	3	NDC recognises Indigenous Peoples' governance over the territory
	4	NDC recognises the legal systems exercised by Indigenous Peoples in their territories
3. Indigenous knowledge systems	1	NDC mentions Indigenous knowledge systems and their inclusion in climate governance at a general level, without elaborating on how this inclusion is operationalised
	2	NDC mentions or values specific practices, based on Indigenous Peoples' knowledge, as a contribution to climate action
	3	NDC elaborates on Indigenous Peoples' worldviews and recognises specific values associated with their knowledge systems – e.g. respect, interdependence
	4	NDC promotes concrete mechanisms for incorporating Indigenous knowledge into specific climate action initiatives
4. Full and effective participation of Indigenous Peoples in climate governance	1	NDC notes in general terms that Indigenous Peoples were involved in the design of the NDC, without providing details on how they were involved
	2	NDC broadly promotes the participation of Indigenous Peoples in climate governance, without proposing specific mechanisms for participation
	3	NDCs note that the participation of Indigenous Peoples during design was carried out through mechanisms specifically dedicated to them
	4	NDC refers to, proposes or establishes concrete mechanisms for the effective participation of Indigenous Peoples in climate governance, including the implementation of action plans
	5	NDC refers to horizontal cooperation with Indigenous Peoples in climate governance, promoting co-creation and collaborative actions
5. References a legacy of colonisation	1	NDC say Indigenous Peoples are vulnerable to climate change
	2	NDC identifies Indigenous Peoples as vulnerable to climate change and also mentions, in general terms, the climatic causes of this vulnerability
	3	NDC proposes concrete mechanisms to address the vulnerability of Indigenous Peoples specifically
	4	NDC recognises the root causes that determine the vulnerability of Indigenous Peoples, with emphasis on processes of colonisation, marginalisation and inequity

Table S2. Levels of recognition by subcategory in first and second submissions (darker blue represents higher scores). Parties organised by region.

Region	Country	Submission	1. Indigenous Peoples as rights-holders	2. Indigenous jurisdiction over land	3. Indigenous knowledge systems	4. Full and effective participation in climate governance	5. References a history of colonisation
Africa	Algeria	First					
	Angola	First					
	Angola	First updated					
	Benin	First					
	Benin	First updated					
	Botswana	First					
	Burkina Faso	First					
	Burkina Faso	First updated					
	Burundi	First updated					
	Burundi	First					
	Cabo Verde	First					
	Cabo Verde	First updated					
	Cameroon	First updated					
	Cameroon	First					
	CAR	First updated					
	CAR	First					
	Chad	First updated					
	Chad	First					
	Comoros	First updated					
	Comoros	First					
	Congo	First					
	Congo	First updated					
	Côte d'Ivoire	First					
	Djibouti	First					
	DR Congo	First					
	DR Congo	First updated					
	Egypt	First					
	Equatorial Guinea	First					
	Eritrea	First					
	Eswatini	First					
	Eswatini	First updated					
	Ethiopia	First					
	Ethiopia	First updated					
	Gabon	First					

	Gambia	First					
	Gambia	Second					
	Ghana	First					
	Ghana	First updated					
	Guinea	First					
	Guinea	First updated					
	Guinea-Bissau	First					
	Guinea-Bissau	First updated					
	Kenya	First					
	Kenya	First updated					
	Lesotho	First					
	Liberia	First					
	Liberia	First updated					
	Madagascar	First					
	Malawi	First					
	Malawi	First updated					
	Mali	First					
	Mali	First updated					
	Mauritania	First					
	Mauritania	First updated					
	Mauritius	First					
	Mauritius	First updated					
	Morocco	First					
	Morocco	First updated					
	Mozambique	First					
	Mozambique	First updated					
	Namibia	First					
	Namibia	First updated					
	Niger	First					
	Niger	First updated					
	Nigeria	First					
	Nigeria	First updated					
	Rwanda	First					
	Rwanda	First updated					
	Sao Tome & Principe	First					
	Sao Tome & Principe	First updated					
	Senegal	First					
	Seychelles	First					
	Seychelles	First updated					
	Sierra Leone	First					
	Sierra Leone	First updated					

	Somalia	First					
	Somalia	First updated					
	South Africa	First					
	South Africa	First updated					
	South Sudan	First					
	South Sudan	Second					
	Sudan	First					
	Sudan	First updated					
	Tanzania	First					
	Tanzania	First updated					
	Togo	First					
	Togo	First updated					
	Tunisia	First					
	Tunisia	First updated					
	Uganda	First					
	Uganda	First updated					
	Zambia	First					
	Zambia	First updated					
	Zimbabwe	First					
	Zimbabwe	First updated					
Asia	Afghanistan	First					
	Armenia	First					
	Armenia	First updated					
	Azerbaijan	First					
	Bahrain	First					
	Bahrain	First updated					
	Bangladesh	First					
	Bangladesh	First updated					
	Bhutan	First					
	Bhutan	Second					
	Brunei	First					
	Cambodia	First					
	Cambodia	First updated					
	China (PRC)	First					
	China (PRC)	First updated					
	Georgia	First					
	Georgia	First					
	India	First					
	Indonesia	First					
	Indonesia	First updated					
	Iraq	First					

	Israel	First					
	Israel	First updated					
	Japan	First					
	Japan	First updated					
	Jordan	First					
	Jordan	First updated					
	Kazakhstan	First					
	Kuwuait	First					
	Kuwuait	First updated					
	Kyrgyzstan	First					
	Kyrgyzstan	First updated					
	Laos	First					
	Laos	First updated					
	Lebanon	First					
	Lebanon	First updated					
	Malaysia	First					
	Malaysia	First updated					
	Maldives	First					
	Maldives	First updated					
	Mongolia	First					
	Mongolia	First updated					
	Myanmar	First					
	Myanmar	First updated					
	Nepal	First					
	Nepal	Second					
	North Korea	First					
	North Korea	First updated					
	Oman	First					
	Oman	Second					
	Pakistan	First					
	Pakistan	First updated					
	Palestine	First					
	Palestine	First updated					
	Philippines	First					
	Qatar	First					
	Qatar	First updated					
	Russian Federation	First					
	Saudi Arabia	First					
	Saudi Arabia	First updated					
	Singapore	First					
	Singapore	First updated					

	South Korea	First					
	South Korea	First (Enhance updated)					
	South Korea	First updated					
	Sri Lanka	First					
	Sri Lanka	First updated					
	Syrian Arab Republic	First					
	Tajikistan	First					
	Tajikistan	First updated					
	Thailand	First					
	Thailand	First updated					
	Timor-Leste	First					
	Turkey	First					
	Turkmenistan	First					
	United Arab Emirates	First					
	United Arab Emirates	Second					
	Uzbekistan	First					
	Uzbekistan	First updated					
	Viet Nam	First					
	Viet Nam	First updated					
Europe	Albania	First					
	Albania	First updated					
	Andorra	First					
	Andorra	First updated					
	Belarus	First					
	Belarus	First updated					
	Bosnia and Herzegovina	First					
	Bosnia and Herzegovina	First updated					
	EU	First					
	EU	First updated					
	Georgia	First updated					
	Georgia	First updated					
	Iceland	First					
	Iceland	First updated					
	Liechtenstein	First					
	Moldova	First					
	Moldova	First updated					
	Monaco	First					
	Monaco	First updated					
	Montenegro	First					
	Montenegro	First updated					

	Norway	First					
	Norway	First updated					
	San Marino	First					
	Serbia	First					
	Switzerland	First					
	Switzerland	First updated					
	The Republic of North Macedonia	First					
	The Republic of North Macedonia	First updated					
	Ukraine	First					
	Ukraine	First updated					
	United Kingdom and Northern Ireland	First updated					
Latin America and the Caribbean	Antigua y Barbuda	First					
	Antigua y Barbuda	First updated					
	Argentina	First					
	Argentina	Second					
	Bahamas	First					
	Barbados	First					
	Barbados	First updated					
	Belize	First					
	Belize	First updated					
	Bolivia	First					
	Bolivia	Second					
	Brazil	First					
	Brazil	First updated					
	Chile	First					
	Chile	First updated					
	Colombia	First					
	Colombia	First updated					
	Costa Rica	First					
	Costa Rica	First updated					
	Cuba	First					
	Cuba	First updated					
	Dominica	First					
	Dominican Republic	First					
	Dominican Republic	First updated					
	Ecuador	First					
	El Salvador	First					
	El Salvador	First updated					
	Grenada	First					

	Grenada	Second					
	Guatemala	First					
	Guatemala	First updated					
	Guyana	First					
	Haiti	First					
	Honduras	First					
	Honduras	First updated					
	Jamaica	First					
	Jamaica	First updated					
	Mexico	First					
	Mexico	First updated					
	Nicaragua	First					
	Nicaragua	First updated					
	Panama	First					
	Panama	First updated					
	Paraguay	First					
	Paraguay	First updated					
	Perú	First					
	Perú	First updated					
	Saint Kitts and Nevis	First					
	Saint Kitts and Nevis	First updated					
	Saint Lucia	First					
	Saint Lucia	First updated					
	Saint Vincent and the Grenadines	First					
	Suriname	First					
	Suriname	Second					
	Trinidad y Tobago	First					
	Uruguay	First					
	Venezuela	First					
	Venezuela	First updated					
North America	Canada	First					
	Canada	First updated					
	USA	First					
	USA	First*					
Pacific	Australia	First					
	Australia	First updated					
	Australia	First updated					
	Cook Islands	First					
	Fiji	First					
	Fiji	First updated					

	Kiribati	First					
	Marshall Islands	First					
	Marshall Islands	Second					
	Micronesia	First					
	Nauru	First					
	Nauru	First updated					
	New Zealand	First					
	New Zealand	First updated					
	Niue	First					
	Palau	First					
	Papua New Guinea	First					
	Papua New Guinea	Second					
	Samoa	First					
	Samoa	Second					
	Solomon Islands	First					
	Solomon Islands	First updated					
	Tonga	First					
	Tonga	Second					
	Tuvalu	First					
	Vanuatu	First					
	Vanuatu	First updated					

Iran, Libya, and Yemen have not submitted an NDC.
